# Adverse childhood experiences, personality traits and internalising disorder among adolescents in Nigeria

**DOI:** 10.4102/sajpsychiatry.v31i0.2423

**Published:** 2025-08-30

**Authors:** Aderonke A. Akintola, Tolulope M. Ogungbemi, Bede C. Akpunne, Taiye E. Ojo

**Affiliations:** 1 Department of Psychology, Faculty of Social Sciences, Redeemer’s University, Ede, Osun State, Nigeria

**Keywords:** adverse childhood experiences, personality traits, internalising disorder, anxiety, depression, adolescents

## Abstract

**Background:**

Internalising disorder, which is characterised by anxiety and depression, is a mental health disorder observed among in-school adolescents in Nigeria, with consequent school dropout, substance use and suicide.

**Aim:**

This study examined the effects of adverse childhood experiences (ACEs) and personality traits on internalising disorder among in-school adolescents in Lagos State, Nigeria.

**Setting:**

The study was conducted among in-school adolescents attending secondary schools in Lagos State, Nigeria.

**Methods:**

A cross-sectional design and multistage sampling techniques were used to select 357 participants (138 males = 38.7%; 219 females = 61.3%) from five high schools in Lagos State, who responded to the ACEs Questionnaire, Personality Inventory and Revised Children’s Anxiety and Depression Scale.

**Results:**

The prevalence of ACEs is 23.8% mild, 33.1% moderate, 13.4% severe experiences being higher on internalising disorder, and 29.7% with no experience. Sixteen per cent of children were clinically significant on internalising behaviour, with 19.3% significant on anxiety and 9% on depression. Adverse childhood experiences significantly predicted internalising disorder; likewise, conscientiousness, neuroticism, and openness to experience consistently and significantly predicted internalising disorder. Adverse childhood experiences and personality traits contributed a significant variance of 16% in anxiety, 26% in depression and a combined 20% to the total variance in internalising disorder.

**Conclusion:**

The study encourages practical, tailored interventions that address ACEs and foster positive personality traits to mitigate against internalising disorder.

**Contribution:**

The study underscores the importance of personality traits in the outcomes of children and adolescents exposed to ACEs in Nigeria.

## Introduction

Internalising disorders are emotional and behavioural disorders. People who have an internalising disorder will keep their problems to themselves or internalise the problems.^1,2^ According to the American Psychiatric Association’s Diagnostic and Statistical Manual of Mental Disorders,^3^ internalising disorders with high levels of negative affectivity include major anxiety disorders and depressive disorders, which are the disorders considered in this study.

Anxiety and depression are internalising disorders that are categories of psychological or mental disorders with symptoms directed inward, and they are conditions dominated by emotionality.^4,5^ According to the American Psychiatric Association’s Diagnostic and Statistical Manual of Mental Disorders,^3,6^ anxiety disorders are psychological disorders that involve dysfunctional reactions to anxiety-inducing situations. It includes excessive fear, uneasiness, worry, apprehension or tension, occurring frequently for at least 6 months, concerning several activities (such as work or school performance or the safety of family members). Anxiety disorder interferes with a person’s capacity to lead a normal life because it deviates from the norm, and could be dangerous to the person in the long run.^4,5^ The disorders are grouped into different categories: generalised anxiety disorder (GAD), panic disorder, social anxiety disorder, among others.^3^ Anxiety disorders are common psychological disorders among adolescents, with a prevalence of about 20% and 15%, respectively,^7^ and they are the most undertreated mental health disorders in adolescents. Studies indicate that about 18% of adolescents with this disorder have been treated.^8^

Depression is a variant of adolescent-onset mood disorders, which is the primary cause of disability and suicide.^9^ A diminished interest usually expresses depression in day-to-day pleasurable activities. It is often characterised by low mood, feelings of inadequacy, low self-esteem, irritability, obvious weight gain or weight loss, a lack of appetite, a lack of sleep or too much sleep, a lack of energy and a lack of concentration.^10^ It affects an estimated 6.7% of adults annually.^9^ Globally, 13% of 10–19-year-olds experience mental disorders, including depression, anxiety and behavioural issues.^10^

Adolescence is the distinctive phase of human development spanning ages 10–19 and marks a transitional period between childhood and adulthood.^10^ It is a critical period when an adolescent is susceptible to anxiety and depression because mental disorders, such as internalising disorders, are influenced by different contributing factors, typically manifesting for the first time.^11^ Anxiety and depression in adolescents can negatively impact their ability to do well in school or work, and can hinder their everyday life functions with consequent degeneration to serious mental disorders.^12^ Cai et al.^13^ suggested that suicidal tendencies, including plans and attempts, are more common in individuals with anxiety and depression – those experiencing suicidality and risk of committing suicide in the future. Internalising disorders (anxiety and depression) are quite prevalent among adolescents.

According to a comprehensive review and meta-analysis, the worldwide estimated prevalence of anxiety from 2000 to 2020 ranged between 8.3% and 27%,^14^ and depression 34%.^14^ In Malawi, one study showed that 21% had anxiety and 22% had depression.^14^ In 2022, Wang et al.^15^ conducted research across Burkina Faso, Ethiopia, Ghana, Nigeria and Tanzania in Africa and reported higher rates of anxiety, with 66.7% of participants experiencing it, and depression, with 41.4% of participants affected. Mbanuzuru et al.^7^ documented a 0.9% incidence of anxiety among children and adolescents worldwide. At the same time, in their study, they recorded 15% anxiety disorders among in-school adolescents in the Southwestern region of Nigeria, using a 12-month time frame and DSM-IV criteria. The prevalence is higher, they claimed, than the 10.1% discovered in research within school-attending adolescents from Anambra State, Nigeria.^7^ The discrepancy is probably influenced by differences in the study design, age range, geographical location and diagnostic standards.^7^ Also, adolescents in Ikere-Ekiti, Nigeria, have a significant prevalence of depression with about 25.5% of in-school adolescents reporting mild to moderate depression, while 14.9% reporting severe depression.^16^ Additionally, Adewuya et al.^17^ from their study carried out in Lagos State, indicated that clinically significant psychotic-like symptoms, including depression, were present in 10.5% of in-school adolescents.^18,19^ Adverse childhood experiences (ACEs) have been linked to exhibiting elevated levels of anxiety and depression among these adolescents.^18,20^

Contributory factors to internalising disorder among these adolescents are ACEs. Adverse childhood experiences are psychologically disturbing events that happen to a child between the ages of 0 and 17 that impact the child’s well-being; they often raise the adolescent’s risk for anxiety and depression.^20^ Adverse childhood experiences encompass different stressful experiences during the early years, such as abuse, neglect and familial dysfunction, which may involve being raised in a household affected by addiction or mental health issues.^20^

Similarly, personality traits, such as the Big Five personality traits examined in this study (conscientiousness, agreeableness, openness to experience, neuroticism and extraversion) which are permanent patterns of ideas, feelings and actions that affect how people behave and react in various circumstances, have been linked to anxiety and depression, and also have been seen as moderating or mediating the relationships between ACEs and the two psychological disorders of anxiety and depression.^21^ It has been described as a trans-diagnostic construct that may have an impact on how symptoms manifest in people with ACE.^22^ Personality qualities, in particular borderline traits, may buffer the relationship between emotional maltreatment in childhood and anxiety symptoms among in-school adolescents to some extent. Maltreatment in childhood may induce depressive symptoms by increasing neuroticism. Personality qualities are moderately heritable and can predict a variety of outcomes, including psychopathology, according to findings from twin and family studies.^23^

Despite the increasing awareness of mental health issues among in-school adolescents in Nigeria and a few studies linking ACEs to anxiety and depression among them, there is a dearth of literature on the interaction of ACEs and personality traits in the prediction of internalising disorder (anxiety and depression) among these in-school adolescents in Nigeria. It is thus hoped that this study will fill that gap.

## Research methods and design

A cross-sectional research design was used to investigate how ACEs and personality traits affect the levels of anxiety and depression in in-school adolescents in Lagos State, Nigeria. The research was conducted in Lagos State, focusing on selected schools in District V, which is divided into four zones: Ajeromi Ifelodun, Amuwo-Odofin, Badagry and Ojo Local Government areas. Additionally, District VI divided into three zones: Ikeja, Mushin and Oshodi Isolo Local Government areas, was included. The schools in these two districts are 11 (junior, secondary and senior secondary).

### Study population

The study population included all the in-school adolescents in Districts V and VI of Lagos State, Nigeria. There are 11 schools in the two districts. However, five schools were selected with a population estimated to be 7015.

### Participants and sampling techniques

Using Yamane’s^24^ formula to calculate the required sample size, a sample size of 378 was calculated for a population of 7015. A multi-stage sampling technique was employed in this study to select a sample size of 378 in-school adolescents, where the secondary schools in Lagos State were already stratified into six districts. A purposive sampling technique was used to choose two of the six districts – V and VI – and simple random sampling technique by balloting was employed to select three out of six schools in District V and two out of five schools in District VI. A proportional sampling technique was then used to determine the sample size of 378 in-school adolescents within the selected schools in the districts. Finally, a systematic sampling technique was adopted in selecting participants in each school. This was done using the school’s register, where students were selected based on multiples of 10 from their serial numbers. Specifically, students with serial numbers 10, 20, 30, 40, 50, and so on, who were willing, were selected from each class to participate in the study. Data from 357 respondents, including 138 males (38.7%) and 219 females (61.3%), with mean age = 14.48, s.d. = 1.94, were eventually deemed valid for the research.

### Instruments

The socio-demographic items include gender, age, parental marital status, family type, family size, and a measure of socioeconomic status: MacArthur Scale of subjective social status.^25^

Adverse Childhood Experiences Questionnaire (ACE-Q) developed by Felitti et al.^26^ was used to measure ACEs. The questionnaire assesses 10 types of childhood trauma, as identified in the ACE Study. Five of these are personal experiences: physical abuse, verbal abuse, sexual abuse, physical neglect and emotional neglect. The remaining five pertain to other family members. Schauss et al.^27^ reported a test–retest reliability of 0.91 for scale. The Big 5 Personality Inventory Questionnaire is a 44-item self-report scale by Vicentini et al.^28^ designed to measure the Big Five personality traits (Openness, Conscientiousness, Extraversion, Agreeableness and Neuroticism). The findings of the research conducted by Dekker and Thiel^29^ indicate that the reliability of personality factors within the OCEAN Model (Openness to Experience, Conscientiousness, Extraversion, Agreeableness and Neuroticism) is relatively high, with Cronbach’s Alpha coefficients ranging from 0.82 to 0.92, suggesting strong internal consistency for these factors. The Revised Children’s Anxiety and Depression Scale (RCADS) devloped by Chorpita et al.^30^ was used to assessed anxiety and depression. The RCADS is a 47-item questionnaire. The Cronbach’s alpha reliability of the RCADS for individuals aged 10–19 is reported as 0.94.^31^

### Procedure

An addressed letter was obtained from the researcher’s institution to the principals of the selected schools to seek their approval to distribute questionnaires to students. The research’s intentions and goals were communicated to the school administration. The school register was used to select the participating students from each class. The selected students were approached in the school hall, and consent forms were provided to them to take home to their parents and guardians. Assent forms were given to the adolescents to fill out. After retrieving the signed consent forms, the students were provided with questionnaires to fill out. The participants and the researcher developed a good connection, and the researcher assured them of the confidentiality of the research while explaining its purpose. The questionnaires were intended to be completed immediately by the participants, while the researcher waited to collect them. Some participants requested incentives before taking part in the study, and the researcher declined their request, fearing it could impact their responses, but gave incentives to those who decided to take part after the questionnaire had been administered. The fieldwork for this research spanned 1 month because of the distant locations of the samples, and a total of 378 copies of the research instruments were administered; 378 were retrieved, whereas 357 were valid. These amount to a 94% response rate.

### Ethical considerations

The researcher strictly adhered to the APA’s (American Psychological Association) guidelines on research demographics ethics. The researcher ensured that participants understood the research’s purpose and that taking part in it was completely voluntary. They were also given the assurance that the information gleaned from this study would be used only for scholarly pursuits. Ethical clearance to conduct this study was obtained from the Redeemer’s University, Directorate of Research Innovation and Partnership (DRIPs) Ethical Committee (No. RUN/REC/2023/106).

## Results

The test of relationship summarised in Table 1 shows that ACEs had a significant positive relationship with depression, *r*(355) = 0.25, *p* < 0.01 and anxiety, *r*(355) = 0.21, *p* < 0.01. This implies that depression and anxiety increase as ACE increases. The test of the relationship between personality traits, depression and anxiety indicated that extraversion had no significant relationship with depression, *r*(355) = 0.04, *p* > 0.05 and anxiety, *r*(355) = 0.10, *p* > 0.05. Agreeableness had a significant negative relationship with depression, *r*(355) = –0.41, *p* < 0.01 and anxiety, *r*(355) = –0.11, *p* < 0.05. Conscientiousness had a significant negative relationship with depression, *r*(355) = –0.34, *p* < 0.01 and anxiety, *r*(355) = –0.19, *p* < 0.01. By implication, as agreeableness and conscientiousness personality traits increase, there also tend to be decrease in the depression and anxiety experienced by students. The relationship between neuroticism and depression (*r*[355] = 0.32, *p* < 0.01) and anxiety (*r*[55] = 0.32, *p* < 0.01) was positively significant. However, openness to experience was significantly related to anxiety positively, *r*(355) = 0.17, *p* < 0.01, but not related to depression, *r*(355) = 0.04, *p* > 0.05.

**TABLE 1:** Correlation matrix showing the relationships among the study variables.

Variables	1	2	3	4	5	6	7	8	9	10	11	12	13
1. Gender	1.00	-	-	-	-	-	-	-	-	-	-	-	-
2. Age	0.03	1.00	-	-	-	-	-	-	-	-	-	-	-
3. Family type	0.08	−0.08	1.00	-	-	-	-	-	-	-	-	-	-
4. Family size	0.04	−0.05	0.28**	1.00	-	-	-	-	-	-	-	-	-
5. Family SES	−0.07	0.12*	0.02	0.07	1.00	-	-	-	-	-	-	-	-
6. ACEs	0.03	0.07	0.18**	0.13*	0.08	1.00	-	-	-	-	-	-	-
7. Extraversion	0.00	0.02	−0.07	−0.05	0.09	−0.01	1.00	-	-	-	-	-	-
8. Agreeableness	−0.02	0.12*	−0.08	−0.10	−0.08	−0.22**	−0.01	1.00	-	-	-	-	-
9. Conscientiousness	0.02	0.06	−0.11*	−0.07	0.03	−0.10	−0.02	0.44**	1.00	-	-	-	-
10. Neuroticism	0.10	−0.03	0.04	0.03	0.04	0.28**	0.04	−0.30**	−0.32**	1.00	-	-	-
11. Openness	−0.03	0.05	−0.05	−0.02	0.03	0.00	0.15**	0.19**	0.24**	0.09	1.00	-	-
12. Depression	−0.03	−0.11*	0.19**	0.08	0.06	0.25**	0.04	−0.41**	−0.34**	0.32**	0.04	1.00	-
13. Anxiety	−0.08	−0.02	−0.00	−0.00	0.08	0.21**	0.10	−0.11*	−0.19**	0.32**	0.17**	0.52**	1.00
Mean	-	14.48	-	6.29	1.79	1.65	25.99	35.65	34.27	22.50	34.42	53.31	61.53
s.d.	-	1.94	-	2.63	1.23	1.62	5.12	5.49	5.89	5.22	5.66	12.57	11.54

Note: *N* = 357;

** , *p* < 0.01;

* , *p* < 0.05; 1, Gender; 2, Age; 3, Family type; 4, Family size; 5, Family SES; 6, ACEs; 7, Extraversion; 8, Agreeableness; 9, Conscientiousness; 10, Neuroticism; 11, Openness; 12, Depression; 13, Anxiety.

ACEs, Adverse Childhood Experiences; s.d., standard deviation; SES, socioeconomic status.

The result also revealed a positive relationship between depression and anxiety, *r*(355) = 0.52, *p* < 0.01. The socio-demographics data indicated that gender had no significant relationship with depression, *r*(355) = –0.03, *p* > 0.05 and anxiety, *r*(355) = –0.08, *p* > 0.05. The relationship between age and depression was negatively significant, *r*(355) = –0.11, *p* < 0.05, implying that the younger adolescents were more affected. In a similar trend, the relationship between family type and depression was found to be significant, *r*(355) = 0.19, *p* < 0.01, indicating that students from polygamous homes tend to experience higher levels of depression compared to those from monogamous homes.

The result in Table 2 indicated that ACEs significantly predicted anxiety (*β* = 0.21, *t* = 4.06, *p* < 0.01). This was with a 4% variance attributed to it. In the next step of the analysis, personality traits were added to the model, and it was observed that extraversion (*β* = 0.06, *t* = 1.23, *p* > 0.05) and agreeableness (*β* = 0.02, *t* = 0.27, *p* > 0.05) were not predictors of anxiety. Conscientiousness predicted anxiety negatively (*β* = –0.15, *t* = –2.61, *p* < 0.01). This means that anxiety decreases with an increase in conscientiousness as a personality trait. On the contrary, neuroticism (*β* = 0.22, *t* = 3.93, *p* < 0.01) and openness (*β* = 0.17, *t* = 3.28, *p* < 0.01) predicted anxiety positively. This implies that students’ experience of anxiety increases with higher levels of neuroticism and openness as personality traits. Jointly, all the variables in Step 2 contributed a significant variance of 16% to the total variance in anxiety *R*^2^ = 0.16, *F*(6, 350) = 11.05, *p* < 0.01, while personality traits added contributed 12% of the variance (Δ*F* = 9.57, *p* < 0.01).

**TABLE 2:** Multiple regression analysis showing adverse childhood experiences and personality traits predicting anxiety.

Variables	*β*	*T*	*P*	*R*	*R* ^2^	Δ*R*^2^	*df*	*F*	Δ*F*
Step 1	-	-	-	0.21	0.04	-	1, 355	16.45**	-
ACEs	0.21	4.06	< 0.01	-	-	-	-	-	-
Step 2	-	-	-	0.40	0.16	0.12	6, 350	11.05**	9.57**
ACEs	0.14	2.70	< 0.01	-	-	-	-	-	-
Extraversion	0.06	1.23	> 0.05	-	-	-	-	-	-
Agreeableness	0.02	0.27	> 0.05	-	-	-	-	-	-
Conscientiousness	−0.15	−2.61	< 0.01	-	-	-	-	-	-
Neuroticism	0.22	3.93	< 0.01	-	-	-	-	-	-
Openness	0.17	3.28	< 0.01	-	-	-	-	-	-

Note: *N* = 357;

** , *p* < 0.01.

ACEs, adverse childhood experiences.

As summarised in Table 3, two-step hierarchical regression was conducted to test the prediction of depression by ACEs and personality traits. In Step 1, depression was regressed on ACEs, and this was significant (*β* = 0.25, *t* = 4.79, *p* < 0.01) in such form, indicating that depression increases with higher levels of ACEs. This was such that it contributed 6% to observed variance in depression. In Step 2, personality traits were added, and the analysis revealed that extraversion was not a significant predictor of depression (*β* = 0.01, *t* = 0.20, *p* > 0.05). Agreeableness negatively (*β* = –0.28, *t* = –5.23, *p* < 0.01) and conscientiousness (*β* = 0.20, *t* = –3.71, *p* < 0.01) negatively predicted depression. This means that depression decreases with an increase in agreeableness and conscientiousness personality traits. On the contrary, neuroticism (*β* = 0.12, *t* = 2.34, *p* < 0.05) and openness (*β* = 0.13, *t* = 2.54, *p* < 0.05) predicted depression positively. This implies that students’ experience of depression increases along with higher levels of neuroticism and openness as personality traits. Jointly, all the variables in Step 2 contributed a significant variance of 26% to the total variance in anxiety *R*^2^ = 0.26, *F*(6, 350) = 20.25, *p* < 0.01, while personality traits that were added in the step contributed to 20% of the variance (Δ*F* = 18.59, *p* < 0.01).

**TABLE 3:** Multiple regression analysis showing adverse childhood experiences and personality traits predicting depression.

Variables	*β*	*t*	*P*	*R*	*R* ^2^	Δ*R*^2^	*df*	*F*	Δ*F*
Step 1	-	-	-	0.25	0.06	-	1, 355	22.90**	-
ACEs	0.25	4.79	< 0.01	-	-	-	-	-	-
Step 2	-	-	-	0.51	0.26	0.20	6, 350	20.25**	18.59**
ACEs	0.13	2.70	< 0.01	-	-	-	-	-	-
Extraversion	0.01	0.20	> 0.05	-	-	-	-	-	-
Agreeableness	−0.28	−5.23	< 0.01	-	-	-	-	-	-
Conscientiousness	−0.20	−3.71	< 0.01	-	-	-	-	-	-
Neuroticism	0.12	2.34	< 0.05	-	-	-	-	-	-
Openness	0.13	2.54	< 0.05	-	-	-	-	-	-

Note: *N* = 357,

** , *p* < 0.01.

ACEs, adverse childhood experiences.

The result summarised in Table 4 indicated that ACEs significantly predicted internalising disorder (*β* = 0.24, *t* = 4.71, *p* < 0.01). This was with a 6% variance attributed to it. In the next step of the analysis, personality traits were added to the model and it was observed that extraversion (*β* = 0.05, *t* = 1.11, *p* > 0.05) and agreeableness (*β* = –0.07, *t* = –1.17, *p* > 0.05) were not predictors of internalising disorder. Conscientiousness predicted internalising disorder negatively (*β* = –0.18, *t* = –3.17, *p* < 0.01). This means that internalising disorder decreases with an increase in a conscientiousness personality trait. On the contrary, neuroticism (*β* = 0.21, *t* = 3.92, *p* < 0.01) and openness (*β* = 0.18, *t* = 3.41, *p* < 0.01) predicted internalising disorder positively. This implies that students’ experience of internalising disorder increases along with increased levels of neuroticism and openness personality traits. Findings in Step 2 showed a contribution of 14% of the variance when personality traits were added (Δ*F* = 14.42, *p* < 0.01). Jointly, all the variables in Step 2 contributed a significant variance of 20% to the total variance in internalising disorder *R*^2^ = 0.20, *F*(6, 350) = 14.42, *p* < 0.01. The result confirmed the hypothesis and it was accepted.

**TABLE 4:** Multiple regression analysis showing adverse childhood experiences and personality traits predicting internalising disorder (anxiety and depression).

Variables	*β*	*t*	*p*	*R*	*R* ^2^	Δ*R*^2^	*df*	*F*	Δ*F*
Step 1	-	-	-	0.24	0.06	-	1, 355	22.16**	-
ACEs	0.24	4.71	< 0.01	-	-	-	-	-	-
Step 2	-	-	-	0.45	0.20	0.14	6, 350	14.42**	12.18**
ACEs	0.15	3.02	< 0.01	-	-	-	-	-	-
Extraversion	0.05	1.11	> 0.05	-	-	-	-	-	-
Agreeableness	−0.07	−1.17	> 0.05	-	-	-	-	-	-
Conscientiousness	−0.18	−3.17	< 0.01	-	-	-	-	-	-
Neuroticism	0.21	3.92	< 0.01	-	-	-	-	-	-
Openness	0.18	3.41	< 0.01	-	-	-	-	-	-

Note: *N* = 357,

** , *p* < 0.01.

ACEs, adverse childhood experiences.

## Discussion

This study explored how ACEs and personality traits contribute to anxiety and depression among in-school adolescents. Findings reveal that while ACEs strongly predict both anxiety and depression, the influence of personality traits varies across these conditions. Neuroticism and openness to experience were found to be strong predictors of anxiety, while conscientiousness also did but in an inverse relationship. Depression was predicted by all the Big Five personality traits except extraversion. Agreeableness and conscientiousness were inversely related to depression; neuroticism and openness to expression had positive linear relationship with the condition. When internalising behaviour (anxiety and depression combined) was regressed on ACEs and personality traits, ACEs, conscientiousness, neuroticism and openness to experience were found to be consistent in predicting these conditions, while extraversion consistently did not across the board.

Scrutinising how the hypotheses with findings were corroborated by literature, the study’s first hypothesis which aimed to ascertain that ACEs and personality traits significantly predict anxiety among in-school adolescents was actually confirmed. Building upon existing literature, the findings align with prior studies emphasising the link between ACEs and anxiety.^32^ Reducing ACEs emerged as a potential strategy to decrease anxiety levels among in-school adolescents, accentuating the significance of early intervention. Personality traits of neuroticism^33^ and openness predicted anxiety; conscientiousness also showed an inverse relationship. This suggests that higher neuroticism and openness to experience are associated with increased anxiety experiences. Extraversion and agreeableness did not play significant roles in internalising behaviour in this study. Various factors, including cultural variations, can influence the complex interplay of the predictive role of personality traits on anxiety. The specific psychological factors prevalent in the studied population could all contribute to the observed differences and significantly impact anxiety and other mental health outcomes. The study by Wu et al.^34^ emphasised that personality traits (i.e. extraversion, agreeableness, neuroticism and openness) could influence anxiety through the chain mediating effects of general self-efficacy and academic burnout. In addition, the research by Okwaraji et al.^35^ focused on the assessment of locus of control, self-esteem and depression among school-going adolescents in Nigerian rural communities.^35^ This study provides insights into the psychological factors that may interact with personality traits to influence mental health outcomes in Nigerian adolescents.

Building on these findings, the study further examined how ACEs and personality traits predict depression among adolescents. Notably, ACEs, neuroticism and openness demonstrated significant positive relationships with depression, while agreeableness and conscientiousness were negative predictors. Extraversion had no significant influence on depression. These results substantiate the notion that an increase in ACEs significantly contributes to elevated depression levels among in-school adolescents. They also indicate that students’ depression levels increase with higher neuroticism and openness personality traits. These findings are in alignment with studies such as those by Elmore and Crouch^36^ and Wu^37^ that reinforce the predictive nature of personality traits, underscoring the importance of considering both ACEs and personality traits in understanding depression.

Finally, the third hypothesis explored whether ACEs and personality traits would predict a joint anxiety and depression disorder, internalising disorder among the in-school adolescents in Lagos State. The results of the analysis support the acceptance of the hypothesis, suggesting that ACEs and specific personality traits play distinct roles in anxiety and depression among students. The finding is consistent with a previous study that suggests that there was a positive association between ACE and impaired personality functioning which is also positively associated with anxiety symptoms, and the interaction between ACE and personality functioning explains a significant amount of variance in anxiety.^22^ Bach et al.^38^ posit that the association between internalising symptoms in adulthood and childhood trauma is mediated by maladaptive personality traits, indicating a major role for these traits in converting the influence of early experiences into later psychopathology. The findings of this study reinforce the importance of a more holistic view of contributory factors of internalising disorder rather than focusing on just one aspect. Examining the roles of personality traits in this study in conjunction with ACEs has given more insight into the dynamics of this disorder, but the study is inexhaustive; other contributing factors could still be researched among the in-school adolescents of Lagos State.

## Limitations

There are some limitations to the study. Since the participants were adolescents aged 10–19, the extensive nature of the questionnaire, comprising 101 questions, may have caused respondent fatigue, impacting the quality of the data collected. Nonetheless, the items of the scales were explained to each group of participants separately to mitigate the impact of fatigue on data quality. Also, given that the study was conducted in just 10 schools (five junior and five senior) and exclusively in government schools, the sample size is small, which limits the generalisability to all adolescents attending schools in Lagos State, Nigeria.

## Recommendations

A multifaceted approach should be implemented to support the mental well-being of in-school adolescents in Lagos State and Nigeria as a whole. Targeted interventions to reduce ACEs should be developed and implemented.

Research revealed that psychoeducational modules focused on resilience-building and coping mechanisms can help reduce the incidence of anxiety and depression,^39^ as these will help to mediate or moderate the effects of ACEs and personality traits. Also, school-based mental health support, including counselling services, awareness programmes and a supportive environment that addresses early signs of anxiety and depression will help.^40,41^ Individualised interventions leveraging the potential of personality traits will go a long way in addressing the problem.^42^ Parents and families should be engaged in mental health programmes to promote open communication within families, fostering resilience, and mitigating the risk of ACEs and internalising disorder.^43,44,45^ Collaboration between schools, mental health workers and community organisations can be established to create a comprehensive support network for adolescents.^46,47^

Educators should be regularly trained in recognising and addressing mental health issues. Teachers should be equipped with tools to create positive and inclusive classroom environment.^48,49^ Therefore, routine mental health assessments should be integrated into the educational system to identify students at risk for timely intervention and support.

## Conclusion

Exploring the intricate connections between personality traits and anxiety revealed a distinctive pattern. While conscientiousness was negatively associated with anxiety, highlighting a potential protective effect, in contrast, neuroticism and openness to new experiences exhibited positive significant relationships with anxiety, indicating an elevated likelihood of anxiety with higher levels of these traits. Likewise, the examination of personality traits and depression displayed noteworthy insights in this study. Extraversion showed no significant relationship with depression and agreeableness, and conscientiousness demonstrated significant negative associations, indicating that higher levels of these traits correspond to lower levels of depression. Conversely, neuroticism and openness to expression exhibited positive significant relationships with depression, suggesting an increased propensity for depression. Much as we know that behavioural genetics and heritability estimates show the contributions of genes in personality traits, the fact that their contributions are not 100%, concerted efforts by parents and stakeholders can be made for improvements in personality traits and also mitigate against ACEs among children and adolescents.
